# What Is Required for the Future of Medical Education and Healthcare?

**DOI:** 10.31662/jmaj.2024-0295

**Published:** 2025-01-31

**Authors:** Takaki Kobayashi, Soichiro Saeki

**Affiliations:** 1 Center Hospital of the National Center for Global Health and Medicine, Tokyo, Japan; 2 Division of Public Health, Department of Social Medicine, Graduate School of Medicine, Osaka University, Osaka, Japan

**Keywords:** Medical Education, Undergraduate, Postgraduate, COVID-19, Japan, Public Health

## Abstract

The COVID-19 pandemic has profoundly transformed medical education, shaping students’ career aspirations and impacting their mental well-being. A global survey among medical students conducted in 2023 revealed that over half of medical and nursing students are inclined toward nonclinical careers, while a substantial majority express concerns regarding their mental health. These insights underscore the urgent need to broaden educational trajectories beyond direct patient care and prioritize the psychosocial welfare of students.

We explore the implications of these shifts, emphasizing the rising interest in public health and research as viable alternative career paths. Medical schools in Japan are increasingly introducing healthcare policy and administration courses, offering students opportunities to assume public health and research roles. Moreover, medical education must adapt to mitigate the pressures associated with future workloads and extensive academic demands by integrating digital technologies, such as artificial intelligence and virtual reality, into the curriculum.

Furthermore, enhancing career guidance and expanding international exchange programs will cultivate a more versatile talent pool and equip students for diverse career trajectories in healthcare. By accommodating clinical and nonclinical interests and addressing student mental health, the future of medical education can elevate healthcare quality and advancing public health outcomes. Ultimately, a holistic and multifaceted approach to medical education is crucial for nurturing professionals adept at confronting the challenges of future pandemics and pioneering innovations in healthcare.

## Introduction: Changes in Medical Students’ Perspectives on Their Careers

The recently published “Clinician of the Future 2023 Education Edition ^(1)^” has meticulously collated findings from an extensive online survey involving 2,212 medical and nursing students from 91 countries. This epistolary endeavor illuminates two pivotal discoveries arising from the survey:

1) Formation of Nonclinical Careers:

“*Globally, 58% of students (54% medical students, 62% nursing students) see their current studies as a stepping-stone toward a broader career in healthcare that will not involve directly treating patients.*”

2) Student Mental Health:

“*Most students globally (60%) are worried about their mental health.*”

More than half of the students were considering careers outside of direct patient care or "clinical" fields, and more than half of the students expressed concerns about their mental health. Considering these discernments, it becomes evident that a compelling imperative exists for augmenting educational avenues associated with nonclinical domains and fortifying mechanisms dedicated to the psychosocial welfare of students.

This study explores the contextual foundations of the prevailing conditions and the resulting survey findings. The seismic impact of the COVID-19 pandemic is undeniably significant ^(2)^. This global health emergency has sharply highlighted the critical importance of not only clinicians―those deeply embedded within internal medicine (including infectious diseases, pulmonology, and related fields), emergency medicine, and intensive care units―but also a diverse array of public health professionals and research scholars across a broad spectrum.

Moreover, in preparation for future pandemics, it is critically important to develop and ensure a multifaceted talent pool. Conversely, the distressing sight of fatigued physicians and nurses working relentlessly in the healthcare sector has undoubtedly provoked students’ concern about the demanding workload and academic rigor necessary for their professional growth. The future medical education system must address the broad talent development needs of society and students, while simultaneously alleviating the root causes of their anxiety.

Before delving into these topics, we clarify that this manuscript reflects the perspectives and initiatives of an institution with a mandate to advance medical practice and research, which may uniquely shape our recommendations. We acknowledge that institutions with different missions and resources may adopt varied approaches to medical education, and our suggestions may particularly resonate with those institutions that are similarly positioned to lead in research and public health administration.

## The Current Status of Medical Education and Medical Students

### 1) The emergence of nonclinical career paths

In addition to clinical practice, public health and research domains are notable alternatives.

#### 1-a) Public health

There has been an increasing trend of universities offering courses on healthcare policy and administrative governance ^(3)^. These scholarly endeavors broaden students’ intellectual horizons, facilitating their prospective envisioning of roles within public health―be it as medical officers. Notably, at the author’s university, aside from the regular curriculum, a selected public health/international health, medical ethics, and healthcare policy/systems course was taught in English.

This pedagogical odyssey engendered profound insights, endowing us with a clear understanding regarding the specific tenets and intrinsic gravitas of the public health domain―a corpus of knowledge that shall indubitably resonate with those who ardently champion the improvement and propagation of public health, as enshrined within the hallowed annals of the national Medical Practitioners’ Act.

#### 1-b) Research

Interest in research careers also appears to be notably strong. In addition to epidemiological research directly related to public health, students demonstrate diverse clinical and basic research interests. Ambitious students can acquire valuable experience by proactively engaging with research laboratories aligned with their passions, whether through conducting studies, participating in research, or contributing to ongoing projects. Integrating opportunities for research exposure into medical education curriculums can serve as a highly effective strategy for nurturing future researchers. At the author’s university, alongside lectures on biostatistics, a well-structured program enabled students to join a research laboratory of their choice for six months during their fourth year of medical school, which proved immensely educational.

### 2) Medical students’ mental health

Concerning the main components of vague anxieties, concerns are raised about forthcoming labor burdens and the profound expanse of erudition required for student scholarly maturation.

#### 2-a) Concerns about future workload

Concerns about future workload naturally arise during routine internships and interactions with senior physicians and nursing staff. As the discourse around the importance of work-life balance intensifies, coupled with the progressive implementation of reforms in the working conditions of physicians in Japan, the adoption of shift systems and the integration of digital technologies are anticipated to enhance the efficiency and effectiveness of daily clinical operations. It is projected that improvements in healthcare professionals’ working conditions will result in an elevated quality of healthcare services, ultimately benefiting patient outcomes and public health ^(4)^.

#### 2-b) Anxiety over the study volume required

Concerns regarding the volume of study required for students are likely addressed through various initiatives undertaken by universities and educational material developers. At the author’s university, prior to Computer-Based Testing in the fourth year of medical school, students were organized into groups of approximately 10 for lectures and exam preparation. This approach naturally cultivated an environment conducive to peer teaching and collaborative learning, thus enhancing educational outcomes. In addition to group-based learning, the integration of tablets and other electronic devices, along with the use of virtual reality technology and artificial intelligence in areas such as anatomy, surgical education, and medical interview practice, indicates that the incorporation of digital technology will play an increasingly pivotal role in the future of medical education ^(5)^.

Despite the limited discussion in the report, the implications of the COVID-19 pandemic on students’ mental health cannot be overlooked. The pandemic may have negatively affected students’ mental well-being by disrupting their routines and learning experiences. Furthermore, witnessing the overwhelming working conditions of healthcare professionals during the pandemic may have heightened students’ apprehension about the demands of their future careers. Additionally, concerns about inadequate training due to pandemic-related restrictions may contribute to feelings of unpreparedness and anxiety, further exacerbating the issues discussed above.

## What Else Can Be Done?

In addition to addressing the diversification of student interests and needs and in light of the need to better prepare for future pandemics, the scope of medical education must extend beyond traditional “lecture content” and “practical experience (internships)” with a healthcare focus. Studies in public health and biostatistics, practical research experiences, internships at public health institutions, and placements within the Ministry of Health are likely to become essential components universities offer to future students.

Moreover, exposure to diverse career paths provides invaluable opportunities. At the author’s university, talk sessions were held approximately once every one to two months, featuring mid-career and veteran professionals from various fields who shared insights into their current roles and career journeys. These sessions followed Q&A segments and discussions on career possibilities with students and faculty. While clinical career paths are easily envisioned through regular internships and lectures, providing students with opportunities to explore the career trajectories of professionals who have excelled in nonclinical fields and to reflect on their career aspirations from an early stage will foster the development of a wide range of talents.

Lastly, the significance of studying abroad warrants attention. At the author’s university, students who passed an internal selection process had the opportunity to participate in research abroad during their fourth year and clinical internships abroad in their sixth year. The author has been privileged to study in the United States and Australia during his academic career. Studying abroad exposes students to different cultures and environments, offering invaluable opportunities for Japanese medical students to broaden their global perspectives and potentially influencing their values in meaningful ways. Expanding such opportunities during student years contributes to the development of specialists in specific fields and cultivates individuals with more expansive worldviews, thus enhancing overall talent development.

## Conclusion: Advancing Medical Education for Diverse Careers and Enhancing Public Health

The future of medical education must equip students for clinical practice and establish and enhance an educational framework that fosters diverse career opportunities in public health, research, and healthcare policy. Additionally, strengthening support systems to mitigate academic pressures and address concerns about the future is critical for addressing student mental health. Leveraging digital technology, expanding study abroad opportunities, and offering career guidance to create an environment where students can excel across various fields in alignment with their interests and abilities should become central objectives of medical education.

In alignment with these goals, the latest “Model Core Curriculum for Medical Education” in Japan provides a significant step forward ^(6)^. By emphasizing the importance of broadening and diversifying medical student and physician career possibilities beyond clinical practice, the curriculum sets a foundation for a more versatile and adaptable healthcare workforce. This forward-thinking approach ensures that future medical professionals are not only prepared to provide clinical care but are also equipped to lead in areas such as public health, research, and policymaking.

Through these initiatives, medical students will be able to access a broader spectrum of career options, enabling the medical field to cultivate a more diverse range of talents. A sustained commitment to advancing medical education will not only elevate the quality of healthcare but contribute to individuals’ overall health and well-being. Innovations in medical education will serve as pivotal steps not only in training healthcare professionals but also in striving for enhanced healthcare services and the progression of public health.

## Article Information

### Conflicts of Interest

None

### Acknowledgement

The authors thank their colleagues, specifically those in the clinical residency training group, for their helpful discussions. The author acknowledges the use of Grammarly (Grammarly Inc., San Fransico, USA) for primary language editing. The views expressed in this manuscript are those of the author and do not necessarily represent the author’s institutions.

### Author Contributions

Both authors conceptualized the manuscript. TK wrote the original draft and SS critically edited it. Both authors have read and approved the final version of the manuscript.

The term “author” in the manuscript refers to TK, and the “author’s university” refers to the Tokyo Medical and Dental University (TMDU, currently Institute of Science Tokyo). TK earned his MD degree from TMDU, while SS earned his MD degree from Osaka University.

### Approval by Institutional Review Board (IRB)

Not applicable.
